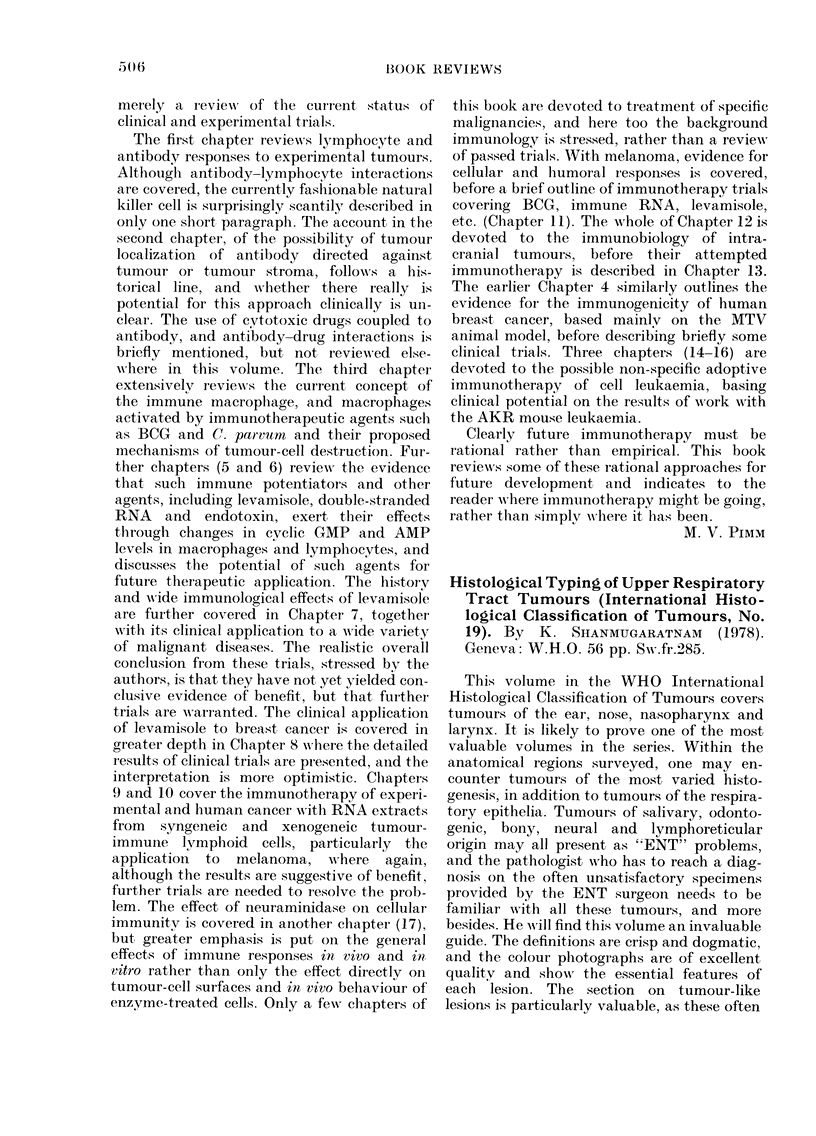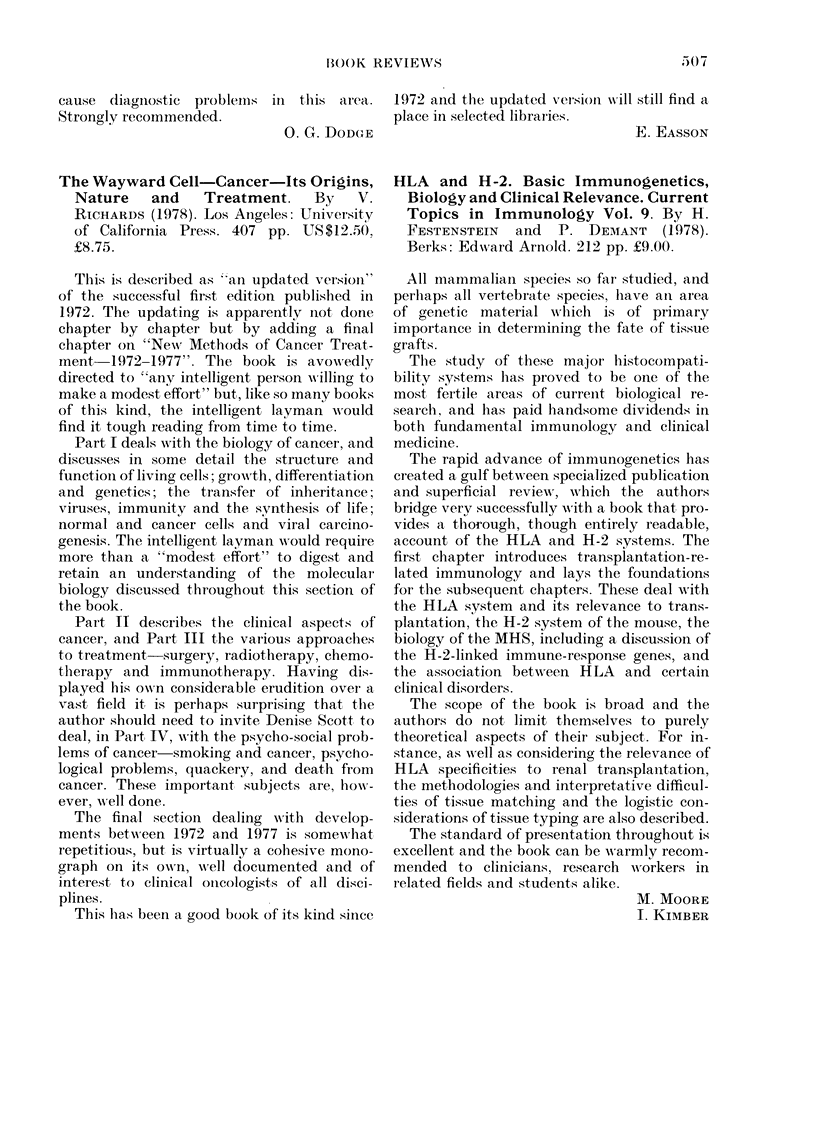# Histological Typing of Upper Respiratory Tract Tumours (International Histological Classification of Tumours, No. 19)

**Published:** 1979-09

**Authors:** O. G. Dodge


**Histological Typing of Upper Respiratory Tract Tumours (International Histological Classification of Tumours, No. 19).** By K. SHANMUGARATNAM (1978). Geneva: W.H.O. 56 pp. Sw.fr.285.

This volume in the WHO International Histological Classification of Tumours covers tumours of the ear, nose, nasopharynx and larynx. It is likely to prove one of the most valuable volumes in the series. Within the anatomical regions surveyed, one may encounter tumours of the most varied histogenesis, in addition to tumours of the respiratory epithelia. Tumours of salivary, odontogenic, bony, neural and lymphoreticular origin may all present as "ENT" problems, and the pathologist who has to reach a diagnosis on the often unsatisfactory specimens provided by the ENT surgeon needs to be familiar with all these tumours, and more besides. He will find this volume an invaluable guide. The definitions are crisp and dogmatic, and the colour photographs are of excellent quality and show the essential features of each lesion. The section on tumour-like lesions is particularly valuable, as these often cause diagnostic problems in this area. Strongly recommended.

O. G. DODGE